# Cholestasis Linked to Bodybuilding Supplements: Exploring the Risks of Contamination

**DOI:** 10.1155/2024/5112461

**Published:** 2024-05-24

**Authors:** Mark Matusak, Jihad Aljabban, Mark Wirtz, Rashmi Agni, Erin Spengler

**Affiliations:** ^1^Department of Family Medicine, University of Wisconsin Hospital and Clinics, Madison, USA; ^2^Department of Medicine, University of Wisconsin Hospital and Clinics, Madison, USA; ^3^Department of Pathology, University of Wisconsin Hospital and Clinics, Madison, USA; ^4^Department of Gastroenterology and Hepatology, University of Wisconsin Hospital and Clinics, Madison, USA

## Abstract

Drug-induced liver injury resulting from herbal and dietary supplement use is increasingly common and underrecognized. We report a case of a 34-year-old male recreational bodybuilder who presented with muscle cramping, fatigue, and diffuse itching in the setting of bodybuilding supplement use. Labs showed cholestatic liver injury, and liver biopsy revealed bland cholestasis and sinusoidal dilation. He was diagnosed with anabolic-androgenic steroid-induced liver injury. He was symptomatically managed with plasmapheresis for debilitating pruritus. This case highlights drug-induced liver injury as a complication of bodybuilding supplement use containing unlabeled anabolic-androgenic steroids. Sports medicine providers should inquire on herbal and dietary supplement use and be aware of potential contaminants.

## 1. Introduction

Cholestasis is defined by derangements in bile acid flow, whether it is from obstruction or a low flow state. Cholestasis can occur intra- or extrahepatically and is caused by several mechanisms, including from drug-induced liver injury or DILI [1]. Diagnosis of DILI requires the exclusion of nondrug factors through history and laboratory assessment [2]. Only a few substances are known to cause characteristic and reproducible patterns of injury. One such agent is anabolic steroids, which may be contained in body-building products [2]. Body builders take supplements to improve muscle mass and strength [3]. Supplements can be purchased the encounter and are ingested orally. Popular supplements include creatine, caffeine, beta-alanine, glutamine, nitric oxide, and choline, among many others [3]. While the benefit of these supplements is up for debate, side effects from these supplements have been described [4–6]. In a national survey, up to 31% of the respondents reported use of bodybuilding supplements [7]. Given its widespread use, it is paramount for clinicians to screen for bodybuilding supplements when confronted with cases of possible anabolic steroid-induced liver injury. We report on a case of suspected bodybuilding supplement contamination resulting in anabolic-androgenic steroid-induced cholestatic liver injury requiring hospitalization and plasmapheresis for severe pruritus.

## 2. Case Report

A 34-year-old man with a history of hepatitis C infection with sustained virologic response (treated with direct acting antivirals) presented to an outpatient clinic with muscle cramping, fatigue, and diffuse itching. He reported dark urine, yellowing of his eyes, and abdominal pain one month prior to presentation. These symptoms self-resolved after two weeks. His only prescription medications were buprenorphine and naloxone for his opioid use disorder and tadalafil for erectile dysfunction. The patient was on said medications for several years. He rarely drank alcohol and denied recreational drug use in the past year. Patient had no diagnosed liver disease. He had a distant history of intramuscular anabolic steroid use but currently only used pre- and postworkout protein supplements that he bought from GNC (General Nutrition Corporation). Patient reports using products from Muscletech.

On presentation, the patient was well-appearing with normal vital signs. He had no jaundice, and his abdomen was soft without tenderness or organomegaly. His muscles were nontender, and the skin exam was normal. Total bilirubin was 2.8 mg/dL, aspartate aminotransferase (AST) was 76 U/L, alanine aminotransferase (ALT) was 191 U/L, and the level of alkaline phosphatase was 103 U/L (normal: 40–150 U/L). INR was 0.9. The creatinine kinase level was 320 U/L. Creatinine, complete blood cell counts, and electrolytes were within normal limits. Evaluation for acute hepatitis A, B, and C was negative. Ceruloplasmin was normal at 33 and we had no further suspicion of Wilson disease. Given symptomatic improvement, the evaluation proceeded on an outpatient basis.

Over the next 10 days, his itching worsened, and he reported a progressive rash over his trunk and extremities. He noted the return of scleral icterus, dark urine, and pale stools. An ultrasound of the liver was normal. Assessments including HIV, TSH, EBV, CMV, and COVID-19 were normal. ANA was negative. The creatinine kinase level was normalized. The total bilirubin had increased from 5.3 mg/dL to 8.2 mg/dL, including a direct bilirubin of 6.6 mg/dL. AST and ALT had decreased, and INR remained normal and GGT was normal at 61. Electrolytes, creatinine, glucose, and albumin were within normal limits. Complete blood cell counts remained normal and no eosinophilia was noted.

**Figure 1:**
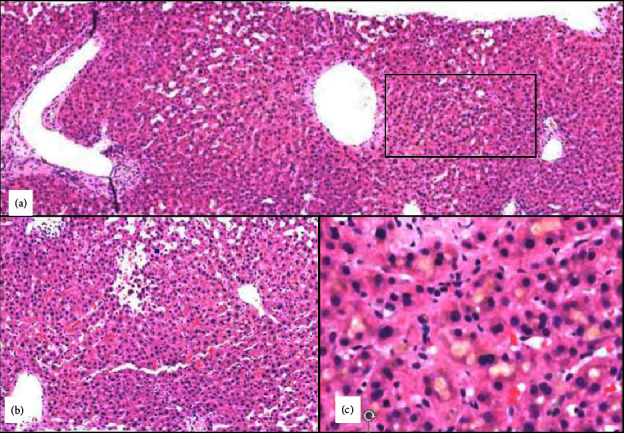
H&E-stained section of liver cores showing azonal sinusoidal dilatation and zone 3 bland cholestasis without significant portal or lobular inflammation or portal bile ductular proliferation. (a-b) Different areas of the core to show diffuse azonal sinusoidal dilatation. (b) Focal peliosis hepatis (cystically dilated spaces communicating with the sinusoids). (c) A high magnification view of the boxed zone 3 area in A showing marked canalicular cholestasis. Bland cholestasis and sinusoidal dilatation/peliosis in isolation are often due to medications and when present together are highly suggestive of anabolic steroid use.

He was admitted to the hospital. Examination was notable for diffuse, erythematous, blanchable macular rash. Scleral icterus and upper abdominal tenderness were present. He did not appear acutely ill but was in distress from severe pruritus. Hepatology and dermatology services were consulted. Additional laboratory workup of smooth muscle antibody, alpha-1 antitrypsin, and mitochondrial antibody were negative. CT scan of the abdomen and pelvis showed no cholecystitis or choledocolithiasis. Liver biopsy demonstrated bland cholestasis and sinusoidal dilation (Figure 1). Over the course of his hospitalization, he had increasingly debilitating pruritus and progressive hyperbilirubinemia to a peak of 8.2 mg/dl. Pruritis was initially treated with cholestyramine, hydroxyzine, cetirizine, diphenhydramine, triamcinolone, and hydrocortisone cream. The patient was started on rifampin and plasmapheresis for three days with marked improvement of pruritis.

Diagnosis of drug-induced liver injury (most consistent with androgen-induced liver injury) was made. At discharge on hospital day five, his total bilirubin decreased to 7.0 mg/dl, other liver function tests normalized, and symptoms had improved. He followed up with hepatology outpatient and felt at his baseline.

## 3. Discussion

Our case highlights an important complication of over-the-counter supplement use. Drug-induced liver injury (DILI) is the leading cause of acute liver failure in Western countries [1]. Herbal and dietary supplements (HDSs) are the second most common cause of idiosyncratic drug-induced liver injury [1, 2, 6, 8–13], with bodybuilding supplements found to be the most common category [9–11]. Anabolic-androgenic steroids have been found in over-the-counter bodybuilding supplements [9, 11–13] and are implicated in a unique phenotype of DILI [1, 10].

Anabolic-androgenic steroid-induced liver injury presents classically with jaundice and severe pruritus [1, 6, 10]. Rash is seen in up to 25% of the cases. Extrahepatic manifestations, including acute kidney injury, have also been noted [8]. Steroid-induced liver injury presents an evolving cholestatic liver injury with progressive elevation in the bilirubin and alkaline phosphatase but with only mild ALT and AST elevations [10, 11, 13]. A liver biopsy demonstrating bland cholestasis with minimal inflammation and no to mild necrosis is typical [1, 6, 10, 11].

The difficult process of making the diagnosis of DILI has been reported at length [2, 8, 11]. Suspicion is a key factor in diagnosis, which can lead to screening for use of implicated substances and withdrawal of concerning agents [2, 6, 9, 10]. Laboratory workup can provide information on the phenotype of injury [2].

Management is primarily symptomatic, as anabolic-androgenic steroid-induced liver injury does not lead to fulminant liver injury, as can be seen with other HDS products [1, 8, 10]. However, the course of symptoms is often prolonged on the order of months [11]. Pruritus may be managed aggressively with bile acid sequestrants [8, 10, 11], rifampin, naltrexone or, as in our case, with plasmapheresis. It is recommended that abnormal labs be followed to resolution [2].

Supplement use in the United States is increasing [10]. Performance or image enhancement is often underlying motivations for use [14]. Therefore, it is important that multidisciplinary sports medicine teams appreciate the implications of over-the-counter supplement use on health, antidoping efforts, and performance [14]. Product contamination will continue to place patients at risk for DILI [9, 11]. Literature shows that 51% of the studied HDSs were mislabeled (chemical analysis showed labels did not accurately reflect actual compounds in products), including 37 of 46 in the appearance and performance enhancing category [9]. Of note, a workout supplement was previously associated with DILI [15]. A study in the UK found 23 of the 24 suspected contaminated supplements contained anabolic steroids [12]. 16 of those supplements declared they contained steroids, but the steroids identified differed from labeling. If liver injury is suspected, the patient should be referred to hepatology. It is important to note that dietary supplements in the United States are considered foods. As such, an implicated product may be reformulated and placed back on the market given the lack of Federal Drug Administration oversight [1, 10].

Contamination presents a unique concern in elite athlete populations where a positive test may lead to disqualification. Well-trained sports medicine providers offering safe, effective, and legal alternatives for performance enhancement may reduce the demand for dangerous, unproven over-the-counter herbal, and dietary supplements. Reporting suspected HDS hepatotoxicity cases and advocating for regulatory change are part of the advocacy work leaders of the healthcare team can perform to improve the health and safety of athletes at all levels and ages [9–11].
